# BTK: a two-faced effector in cancer and tumour suppression

**DOI:** 10.1038/s41419-018-1122-8

**Published:** 2018-10-18

**Authors:** Miran Rada, Nickolai Barlev, Salvador Macip

**Affiliations:** 1 Department of Surgery, McGill University Health Center Research Institute, Cancer Research Program, Montreal, Quebec Canada; 2 Department of Biology, School of Science, Faculty of Science and Education Sciences, University of Sulaimani, Sulaimaniyah, Kurdistan Region Iraq; 3 Institute of Cytology, RAS, Saint-Petersburg, Russia; 4 Cell Signaling Laboratory, Moscow Institute of Physics and Technology, Dolgoprudnoye, Moscow Region Russia; 5 Mechanisms of Cancer and Aging Laboratory, Department of Molecular and Cell Biology, University of Leicester, Leicester, UK

## Abstract

Many genes of the human genome display pleiotropic activity, playing an important role in two or more unrelated pathways. Surprisingly, some of these functions can even be antagonistic, often letting to divergent functional outcomes depending on microenviromental cues and tissue/cell type-dependent parameters. Lately, the Bruton’s tyrosine kinase (BTK) has emerged as one of such pleiotropic genes, with opposing effects in cancer pathways. While it has long been considered oncogenic in the context of B cell malignancies, recent data shows that BTK can also act as a tumour suppressor in other cells, as an essential member of the p53 and p73 responses to damage. Since BTK inhibitors are already being used clinically, it is important to carefully review these new findings in order to fully understand the consequences of blocking BTK activity in all the cells of the organism.

The antineoplastic mechanisms that protect cells from malignant transformation form an intricate network that involves hundreds of proteins working in exquisite coordination. The classic view considers that these tumour suppressors oppose the effects of pro-oncogenic signals, providing a ying-and-yang style equilibrium that, if perturbed, can lead to tumorigenesis. However, the distinction between “good” and “bad” proteins, although convenient to simplify a very complicated landscape, is often inaccurate, since many of them are able to play divergent roles depending on the context. One of such proteins is the Bruton’s tyrosine kinase (BTK), which has recently been shown to have a crucial role in tumour suppression pathways, despite its well-characterized oncogenic activity in blood malignancies.

BTK is a Tec family kinase present at the cell membrane as well as the nucleus, and plays an essential role in B cell maturation as part of the B cell receptor (BCR) signalling pathway, regulating cellular processes such as differentiation and signalling^1^. Because of its importance in B cell physiology, the inherited mutations of BTK that have been found in humans can lead to an immunodeficiency state called agammaglobulinemia^2^. BTK is able to phosphorylate both serines and tyrosines within its target substrates, and in B cells BTK is activated after antigen binding to BCR, which leads to its phosphorylation at tyrosine 551 by SRC family kinases and its autophosphorylation at tyrosine 223^3^. This activates BTK and triggers a cascade that leads to the induction of pro-survival and proliferative signals essential for B cell activity.

Apart from its physiological functions, it has been known for a long time that BTK is highly expressed in B cell malignancies, such as chronic lymphocytic leukaemia, mantle cell lymphoma, and multiple myeloma. Therefore, BTK displays oncogenic activity in these diseases and it was proposed that blocking BTK could have a therapeutic impact^4^. This has led to the development of chemical inhibitors of BTK that have shown a strong effect, thus changing drastically the way these conditions are currently managed^4^. For instance, ibrutinib, a small-molecule inhibitor that forms a covalent bond with BTK near the ATP binding site at Cysteine 481 and blocks its autophosphorylation, has shown efficiency against different B-cell malignancies and has already been approved for clinical use^5^. Other more specific inhibitors are already undergoing clinical trials^6^.

The complexity of BTK functions is just beginning to emerge and several papers indicate that, depending on the context, BTK would not be oncogenic but would contribute to tumour suppressor pathways instead. It has been reported that BTK can induce cell death in several models through mechanisms not clearly understood, which suggested that BTK had functions not only in blood cells but also in other cell types. However, it was not immediately clear what the activity of BTK could be outside the BCR pathway. We identified BTK in a screen of proteins selectively upregulated in senescence of epithelial cells^7^. This underscored the pleiotropic nature of BTK and suggested that BTK could be involved in the cellular responses to damage. Indeed, our investigations eventually characterized BTK as a novel member of the p53 pathway.

p53 is one of the most potent tumour suppressors and it is tightly controlled in the cell. Being a transcription factor, p53 regulates the induction of both coding and non-coding genes that aid to the establishment of cell cycle arrest, senescence and/or apoptosis^8^. A fundamental regulatory mechanism of p53 is mediated by the nuclear export and proteasome-mediated degradation of the polyubiquitinated protein^9^. Phosphorylation of p53 at different residues after DNA damage, disrupts the ubiquitination of p53 by the ubiquitin ligase MDM2, thus stabilizing p53 protein levels^10^. This phosphorylation is performed by a varied range of kinases that respond to different stress signals, including ATM, ATR, DNA-PK, Chk1 and Chk2^11^. We found that BTK can also be included in this group. BTK levels increased after damage in a p53-dependent manner and then it phosphorylated p53 at several residues at the N-terminus, mainly at serine 15^12^. This post-translational modification stabilized p53 and thus modulated its activity, increasing the strength of its cellular responses. Specifically, apoptosis and senescence were greatly influenced by the expression of BTK, up to the point that BTK downregulation with chemical inhibitors or genetic knockdown significantly impaired p53 functions. The fact that p53 also increased BTK levels suggested that the relationship between these two proteins is regulated by a positive feedback loop, with the ultimate goal of reinforcing p53 activity in damage responses.

We proposed that the BTK-dependent phosphorylation of p53 disrupted the p53-MDM2 interaction and subsequent ubiquitination. However, the impact of BTK on the p53 pathway was eventually found to be more profound. We showed that BTK also binds to and phosphorylates MDM2, which leads to a loss of ubiquitination activity and further stabilisation of p53^13^. BTK increased the protein levels of MDM2, consistent with the fact that MDM2 is a p53 target gene. However, the ability of MDM2 to ubiquitinate p53 or itself was diminished in the presence of BTK, which shows that although protein levels were elevated, MDM2 activity was blocked by the BTK-dependent phosphorylation. This inhibition of the ubiquitination functions could be the result of BTK phosphorylating MDM2, although a BTK-mediated phosphorylation of p53 could have an indirect effect as well. Further experiments will be needed to determine whether BTK can phosphorylate MDM2 directly or there are indirect mechanisms involved.

It is known that BTK can trigger cell death and enhance it after damage independently of its effects on the p53 pathway, which suggested that its role as tumour suppressor was not limited to stabilizing p53. We uncovered that this could be in part mediated by the fact that BTK can also increase p73 protein levels^14^. Since p73 shares many functions with p53^15^, this suggests that BTK could be enhancing p73 activity as a fail-safe mechanism to supplement or even substitute p53 functions when needed.

In summary, it is now clear that BTK has two distinct and opposed functions that are context dependent, either increasing proliferation and survival signals or inducing apoptosis and senescence (Fig. 1). It will be important to carefully consider the different activities of BTK when using inhibitors in the clinic, since blocking tumour suppressor pathways could be an undesired side effect. So far, there have been no reports of increased incidence of secondary malignancies in patients with leukaemia treated with BTK inhibitors, but the complexity of its functions suggests that it is a possibility that should be carefully considered.

**Fig. 1:**
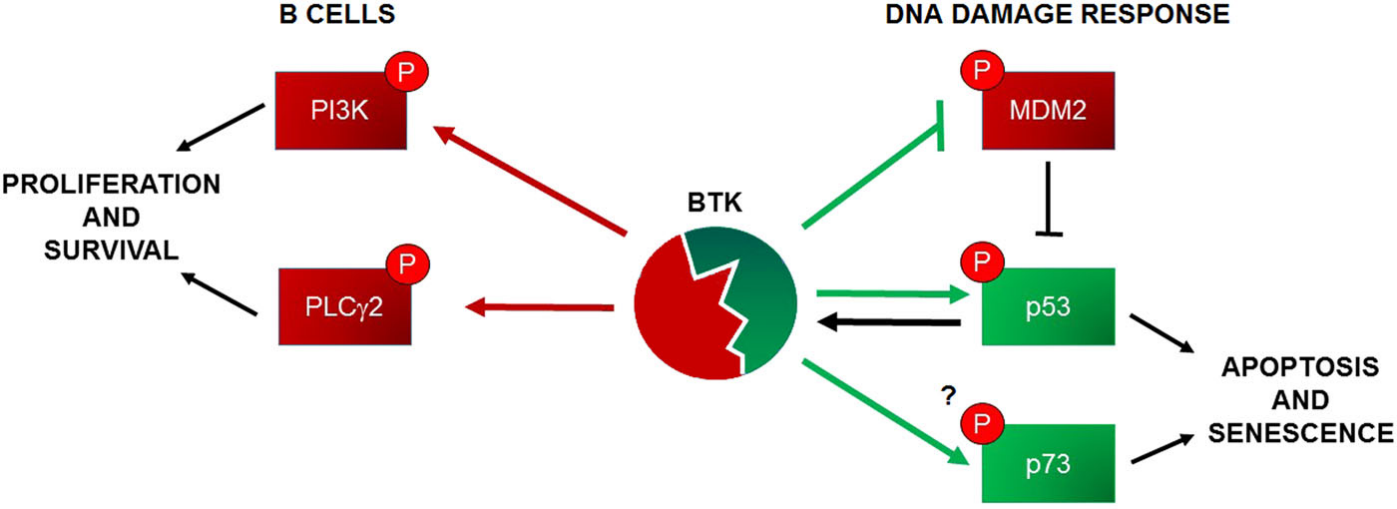
The pleiotropic roles of BTK in cancer. Schematic representation of the involvement of BTK in tumour suppressor (in green) and pro-oncogenic pathways
